# Test–Retest Reliability of Single-Arm Closed Kinetic Chain Upper Extremity Stability Test

**DOI:** 10.3390/jfmk11010046

**Published:** 2026-01-21

**Authors:** Andy Waldhelm, Mareli Klopper, Matthew Paul Gonzalez, Stephanie Flynn, Edward Austin, Ron Masri

**Affiliations:** 1 School of Rehabilitation, South College, 400 Goody Lane, Knoxville, TN 37922, USA; 2 Department of Physical Therapy, University of South Alabama, 5121 USA Dr. North, Mobile, AL 36688, USA; 3 Department of Physical Therapy, Graceland University, 1401 W. Truman Rd, Independence, MO 64050, USA; klopper1@graceland.edu; 4 Department of Kinesiology, University of Texas at San Antonio, One UTSA Circle, San Antonio, TX 78249, USA; matthew.gonzalez2@utsa.edu; 5 Health Motion Physical Therapy, 3826 44th Street SE, Grand Rapids, MI 49512, USA; skanine@south.edu; 6 Ochsner Health System, 1221 S Clearview Pkwy, New Orleans, LA 70121, USA; edward.austin@ochsner.org; 7 Total Motion Physical Therapy, 757 University City Blvd, Blacksburg, VA 24060, USA

**Keywords:** return to sport, movement systems, upper extremity injury, limb symmetry

## Abstract

**Background:** The original Closed Kinetic Chain Upper Extremity Stability Test (CKCUEST) is a simple assessment tool but does not account for individual differences in hand starting position and fails to provide information on limb asymmetries. The purpose of the study is to evaluate the test–retest reliability of a new single-arm CKCUEST as well as the reliability of the limb symmetry index (LSI). This version normalizes the test based on the participant’s arm length and allows for the assessment of limb symmetry since it is performed one arm at a time. **Methods:** Twelve healthy young adults provided both verbal and written consent to participate. Participants were excluded if they had sustained an injury in the past three months requiring medical attention and/or resulting in decreased activity for more than three days. Testing was conducted in the push-up position with participants’ thumbs placed parallel and at a distance equal to the length of their dominant arm (measured from the acromion to the tip of the middle finger), and feet positioned shoulder-width apart. Participants were instructed to keep the testing hand stable on the floor while the opposite hand reached across the body to touch the stationary hand and then return to the starting position marked with athletic tape. The goal was to complete as many touches as possible in 15 s, with each touch counted only if the participant touched the stationary hand, returned to the starting position, and maintained the shoulder-width stance. The average number of touches from the three trials was used for analysis. Intraclass Correlation Coefficients (ICC_(3,1)_) were computed to determine test–retest reliability. **Results:** Test–retest reliability of the single-arm CKCUEST individual tests was good to excellent. The ICC_(3,1)_ was 0.88 (95% CI: 0.74–0.95) for all tests, 0.89 (95% CI: 0.66–0.96) for the dominant arm, and 0.93 (95% CI: 0.78–0.98) for the non-dominant arm. In contrast, the reliability of the Limb Symmetry Index (LSI) was questionable, showing substantial variability with an ICC_(3,1)_ of 0.53 (95% CI: −0.03–0.83) between Day 1 and Day 2, despite similar mean values (Day 1: 93.6 ± 8.46; Day 2: 94.8 ± 5.77). The Kappa coefficient suggested a substantial level of agreement for the direction of the asymmetry (preferred limb) (Kappa coefficient = 0.62). **Conclusions**: The new single-arm CKCUEST, which personalizes the hand starting position and measures limb symmetry, demonstrates high reliability among healthy young adults.

## 1. Introduction

Upper extremity injuries constitute approximately 30% of sports-related injuries [1,2]. Specifically, shoulder injuries account for 16–35% of musculoskeletal injuries in athletes participating in overhead sports, such as baseball, softball, swimming, and water polo [3,4,5]. The consequences of these injuries are multifaceted, potentially affecting future athletic performance and resulting in long-term health issues. There is also a potential economic burden, including the cost of medical care and lost income for professional athletes. Additionally, there is an economic burden, including medical costs and lost income for professional athletes. Psychologically, these injuries can lead to depression, anxiety, and a fear of reinjury [6]. Notably, reinjury rates can be high, with up to 64% of athletes with shoulder instability [7] experiencing reinjury and up to 26% of athletes suffering additional elbow injuries after ulnar collateral ligament reconstruction [8].

Physical performance tests that assess an athlete’s readiness to return to sport (RTS) have been shown to reduce reinjury rates in athletes recovering from lower extremity injuries when specific criteria are met [9,10,11,12]. However, research to guide RTS decision-making for upper extremity injuries is limited, and no consensus exists on the best practices for this process [13,14]. Currently, decisions about an athlete’s readiness to return are often based on subjective reports, strength, and range of motion (ROM) measurements, with time since injury or surgery being the most commonly used criterion for return to play in upper extremity injuries [15]. A retrospective case–controlled study found that athletes following a return-to-sport testing protocol had a lower rate of recurrent shoulder instability compared to those cleared based on time from surgery [16]. Similarly, a case series using an upper extremity functional testing algorithm reported no additional glenohumeral subluxations, dislocations, or upper extremity injuries requiring surgical intervention at a six-year follow-up [17]. Using predetermined criteria for RTS can reduce reliance on subjective judgment and improve decision-making [18].

For performance tests to be clinically useful, it must demonstrate acceptable test–retest reliability, reflecting true performance differences rather than measurement error. Reliable upper extremity assessments are particularly important for monitoring progression over time, informing return-to-sport or work decisions, and supporting research reproducibility. Establishing reliability in physically active individuals is essential, as this population is frequently evaluated in clinical, industrial, and sports medicine settings.

Functional tests are frequently used in RTS assessments. The Closed Kinetic Chain Upper Extremity Stability Test (CKCUEST) is popular due to its simplicity, low cost, and ability to measure upper extremity stability, speed, endurance, and strength [19]. The original CKCUEST, described by Goldbeck and Davies [20], is a reliable functional test but has limitations. It uses a fixed distance for hand placement, which does not account for individual differences in arm length, and it does not provide a limb symmetry index as both arms are used simultaneously. These limitations may affect its ability to identify individuals at risk of injury or those currently injured based solely on the number of touches [19,20,21]. While there have been studies exploring modified positions for the CKCUEST [22,23,24,25] due to a positive correlation with arm length [24], these modifications still do not address limb symmetry.

Therefore, the purpose of this study was to examine the test–retest reliability of a novel single-arm CKCUEST and the associated limb symmetry index (LSI) in physically active individuals. This modified approach accounts for individual arm length and allows for unilateral assessment of upper extremity performance. The hypothesis is that the new single-arm CKCUEST will exhibit excellent test–retest reliability and good reliability of the LSI.

## 2. Materials and Methods

### 2.1. Research Design

This cross-sectional reliability study was conducted to examine the reliability of the newly developed single CKCUEST. In accordance with methodological recommendations for reliability studies, all testing was performed in a standardized, controlled environment to minimize external variability and measurement error. Data collection occurred indoors in a closed setting (a college classroom) to ensure consistency across testing sessions.

All measurements were collected by a single rater, the primary investigator, who has 22 years of clinical experience as a licensed physical therapist. The use of a single, experienced examiner was intentional and aligns with classical test theory principles, which emphasize controlling rater variability to enhance internal consistency and reliability when evaluating a novel assessment instrument.

### 2.2. Participants

A convenience sample of 12 healthy college students (9 males, 3 females) participated in this study (see Table 1 for demographics). Participants were included if they were between 18 and 33 years of age, reported engaging in regular physical activity—including resistance training—at least once per week for a minimum of six months prior to enrollment, and were able to perform closed kinetic chain upper extremity tasks without pain or limitation. All participants had no prior experience with this specific assessment.

Participants were excluded if they reported any upper extremity injury within the previous three months that required medical attention or resulted in decreased physical activity for more than three days; a history of upper extremity surgery; current pain during upper extremity weight-bearing activities; or any neurological or musculoskeletal condition that could affect upper extremity function.

This convenience sample was selected as an initial step in the development of the assessment, with the intent of establishing test–retest reliability in a healthy, physically active population prior to application in injured or athletic populations. Prior to testing, informed verbal and written consent were obtained, and participant demographics, recent injury history, and dominant-arm length were recorded. An a priori power analysis (power = 0.80, α = 0.05), using pilot data from five participants and assuming an intraclass correlation coefficient (ICC_(3,1)_) of 0.90, indicated that a minimum of 11 participants was required.

The study was conducted in accordance with the Declaration of Helsinki, and approved by the Institutional Review Board of the University of South Alabama (protocol code 21-389 and date of approval: 13 October 2021).

### 2.3. Procedures

**Figure 1:**
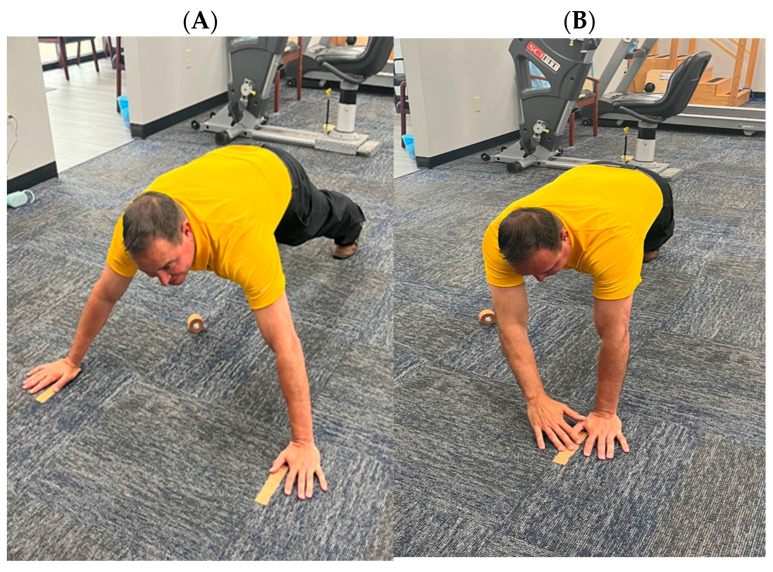
Single-Arm Closed Kinetic Chain Upper Extremity Stability Test: (**A**) Starting and ending positions, (**B**) Middle position.

Participants completed a self-directed warm-up lasting three to five minutes, which included, but was not limited to, arm circles and arm swings in flexion/extension and horizontal abduction/adduction, before performing the single-arm CKCUEST. The test followed a protocol similar to the original CKCUEST [20]. Participants executed the test in a push-up position with their thumbs placed parallel and at a distance equal to the length of their dominant arm (measured from the acromion to the tip of the middle finger), with feet positioned shoulder-width apart (Figure 1A). They were instructed to keep their testing hand stable on the floor while the other hand reached across their body to touch the stationary hand (Figure 1B) and then return to the starting position, marked with athletic tape (Figure 1A). This method differs from the original test, which required alternating arms and recorded each touch as a repetition.

In the new protocol, participants performed the movement as quickly as possible for 15 s, with each return to the starting position counted as one repetition. Repetitions were not counted if the participant did not touch the stationary hand, failed to return to the starting position, moved their feet from shoulder width apart, or did not remain on their toes. Each participant completed one practice trial and three test trials with each hand, alternating which hand was stationary. A one-minute rest period was provided between trials. The average number of repetitions from the three test trials for each arm was used for statistical analysis.

### 2.4. Statistical Analysis

To determine the test–retest reliability of the single-arm CKCUEST, ICC_(3,1)_ values were calculated. The ICCs were calculated for the dominant arm, non-dominant arm, both arms combined, and the LSI values. To determine the direction of the asymmetry (preferred limb), an “IF function” was utilized as described by Bishop and colleagues [25]. Kappa coefficients were calculated to determine the level of agreement for the direction of asymmetries within and between sessions. Kappa coefficients were interpreted as poor (<0.01), slight (0.01–0.20), fair (0.21–0.40), moderate (0.41–0.60), substantial (0.61–0.80), and almost perfect (0.81–0.99) [26]. A *p*-value of 0.05 was used for all statistical analyses.

## 3. Results

Descriptive statistics for the single-arm CKCUEST for dominant and non-dominant arm on Day 1 and 2 are presented in Table 2. Test–retest reliability of the single-arm CKCUEST individual tests was good to excellent. The ICC_(3,1)_ was 0.88 (95% CI: 0.74–0.95) for all tests, 0.89 (95% CI: 0.66–0.96) for the dominant arm, and 0.93 (95% CI: 0.78–0.98) for the non-dominant arm. In contrast, the reliability of the Limb Symmetry Index (LSI) was questionable, showing substantial variability with an ICC_(3,1)_ of 0.53 (95% CI: −0.03–0.83) between Day 1 and Day 2, despite similar mean values (Day 1: 93.6 ± 8.46, 95% CI: 88.2–98.9; Day 2: 94.8 ± 5.77, 95% CI: 91.2–98.5). Kappa coefficient suggested a substantial level of agreement for the direction of the asymmetry (preferred limb) (Kappa coefficient = 0.62).

## 4. Discussion

The objective of this study was to assess the reliability of the new single-arm CKCUEST. The results demonstrated good to excellent test–retest reliability, partially supporting the study hypothesis. In contrast, the reliability of the LSI derived from the single-arm CKCUEST was poor, leading to rejection of that portion of the hypothesis. These findings highlight the importance of distinguishing between relative reliability of a test and the variability inherent in derived asymmetry measures.

Recent literature has emphasized the need for standardized return-to-sport (RTS) testing to reduce reinjury risk following upper extremity injury [13,18]. The original CKCUEST has been shown to be a reliable and valid measure of upper extremity strength and stability [20]; however, it has notable limitations related to test standardization. Specifically, the fixed distance between markers does not account for individual differences in arm length, which has been shown to significantly influence CKCUEST performance and limit between-subject comparisons [24]. To address this limitation, the single-arm CKCUEST was developed using the participant’s arm length to standardize marker placement, thereby improving protocol consistency and reducing measurement error associated with anthropometric variability.

An additional limitation of the original CKCUEST is the alternating use of both upper extremities, which precludes direct comparison between limbs. Given that RTS decision-making frequently relies on comparisons between the involved and uninvolved limbs [27], the single-arm CKCUEST allows for unilateral testing and direct limb-to-limb comparison. Furthermore, the single-arm format may impose greater postural and neuromuscular demands due to increased time spent in single-limb support, potentially enhancing its sensitivity to upper extremity stability deficits.

The reliability of the single-arm CKCUEST (ICC: 0.88–0.93) was comparable to that of the original CKCUEST (ICC: 0.79–0.97) [20,28,29]. Additionally, the single-arm CKCUEST showed similar reliability to modified versions of the CKCUEST, where hand positions were adjusted to one-half arm length (ICC: 0.88–0.93 vs. ICC: 0.92) [22] or positioned directly under the chest (ICC: 0.88–0.93 vs. ICC: 0.79–0.93) [23,25].

Work by Belmonte and colleagues has highlighted that high relative reliability values (e.g., ICC) may coexist with substantial measurement variability when test standardization or familiarization is limited [30]. In such cases, individuals may maintain consistency between sessions, resulting in high ICC values, despite meaningful within-subject variability across repeated measurements. Although the present study employed a standardized protocol, only a brief familiarization period consisting of one practice trial was provided, which may have contributed to increased absolute variability, particularly for the LSI. This distinction between relative reliability and measurement variability is clinically important, as return-to-sport decision-making often relies on absolute thresholds or asymmetry scores rather than consistency. Therefore, while the single-arm CKCUEST demonstrates strong relative reliability, clinicians should interpret asymmetry results with caution and consider incorporating repeated testing, multiple trials, or series of assessments a when making return-to-sport decisions.

From a clinical perspective, the LSI is appealing due to its simplicity and frequent use as a benchmark in return-to-sport decision-making; however, the present findings highlight important limitations when applying LSI values at the individual level. Because the LSI is a ratio-based measure, small absolute changes in unilateral performance may be magnified, leading to unstable or misleading asymmetry values across sessions. Consequently, reliance on a single LSI score to guide clearance decisions may overestimate clinically meaningful differences between limbs. Instead, LSI values derived from the single-arm CKCUEST should be interpreted in conjunction with absolute performance measures, movement quality, and trends across repeated testing sessions. This multifactorial approach may improve clinical decision-making and reduce the risk of misclassifying an individual as ready to return to sport.

Most studies do not report the reliability of the LSI, particularly for upper extremity function and return-to-sport testing. The findings of the present study were comparable to other upper extremity assessments, including the prone medicine ball drop test in the 90°-90° position, the prone medicine ball drop test in the 90° position, and the half-kneeling medicine ball rebound test [31], but demonstrated lower reliability than the seated single-arm shot put test [31,32]. Although intersession differences in single-arm CKCUEST LSI were smaller than those reported for other tests [31,32], the larger standard deviations and confidence intervals observed in this study indicate greater within-subject variability.

Bailey and colleagues have highlighted the need for reliability analysis for asymmetry measures as the lack of this analysis may bias interpretations of the interlimb asymmetries [33]. Similarly, Bishop has also highlighted that noise may be introduced when using ratio data (such as the limb symmetry index) which may affect the measurement error of the data [34]. As such this study sought to quantify the reliability of the LSI measures of the CKCUEST. While the kappa coefficients highlighted substantial agreement for the direction of the asymmetry (which limb was the preferred limb) the magnitude of the symmetry (percent difference between limbs) had high variability (95% CI = −0.03–0.83). As such, while the CKCUEST may be good test to determine which limb is being favored, caution should be utilized when determining how much of a difference between limbs is present. These findings are consistent with the few studies that have examined the reliability of asymmetry measures in various assessments. Xu and colleagues have found high variability in asymmetry measures derived from the countermovement jump, drop jump, and rebound jump (coefficient of variation = 20.74–38.51%) [35]. Similarly, Perez-Castilla and colleagues found low reliability of interlimb asymmetries during the unilateral and bilateral broad jump (ICC = −0.40–0.58) [36]. Overall, this suggests that practitioners should be cautious when examining interlimb asymmetry research, particularly those studies that do not examine their reliability.

### 4.1. Limitations

The single-arm CKCUEST was found to be a reliable assessment tool; however, this study had several limitations. The sample consisted of a small number of participants, all of whom were healthy graduate-level college students, with an uneven sex distribution. These factors limit the generalizability of the findings to other populations.

### 4.2. Future Studies

This study represents the initial reliability investigation of the newly developed single-arm CKCUEST. Future research should include larger and more diverse samples across a wider range of age groups to enhance generalizability. Additionally, studies should examine the reliability of the test in various athletic populations, including both injured and uninjured individuals. Finally, validity studies are warranted to determine whether the single-arm CKCUEST can be used to identify individuals at increased risk for injury or to inform return-to-sport decision-making following injury.

### 4.3. Practical Application

This study introduces the single-arm CKCUEST, which normalizes hand placement to individual arm length, and demonstrates excellent test–retest reliability in healthy young adults. Unlike the original test, this version permits assessment of limb symmetry, although the limb symmetry index demonstrated only questionable reliability. These findings support the single-arm CKCUEST as a reliable measure of upper extremity stability while underscoring the need for further research on its role in return-to-sport assessment.

## 5. Conclusions

Results indicate that the new single-arm CKCUEST demonstrates good to excellent test–retest reliability for individual tests while the LSI reliability was questionable among healthy young adults. Further research is needed to establish its utility in identifying individuals at risk for injury or determining readiness to return to sport following an upper extremity injury. The primary advantage of this test is that it allows clinicians to tailor the original CKCUEST setup to the individual’s anthropometric measurements. Additionally, the side-to-side comparison provides valuable information on limb symmetry, which can be particularly useful in return-to-sport assessments, especially in populations lacking normative values.

## Figures and Tables

**Table 1:** Descriptive statistics (Mean and SD) of participants.

	Total (*n* = 12)	Males (*n* = 9)	Females (*n* = 3)
Age	23.6 ± 4.64	22.8 ± 3.87	26.0 ± 7.00
Height (cm)	179 ± 18.2	181 ± 6.73	172 ± 8.15
Weight (kg)	79.7 ± 17.5	84.2 ± 17.1	66.5 ± 13.0
Arm length (cm)	76.8 ± 4.52	76.5 ± 4.47	71.9 ± 2.92
Right-hand-dominant	11	8	3

**Table 2:** Descriptive statistics (Mean and SD) for the single-arm CKCUEST for Day 1 and 2.

	Dominant	Non-Dominant
Day 1 (repetitions)	24.33 ± 3.69	26.19 ± 4.54
Day 2 (repetitions)	27.13 ± 4.17	28.72 ± 4.82

## Data Availability

The original contributions presented in this study are included in the article. Further inquiries can be directed to the corresponding author.
